# Senescent cells evade immune clearance via HLA-E-mediated NK and CD8^+^ T cell inhibition

**DOI:** 10.1038/s41467-019-10335-5

**Published:** 2019-06-03

**Authors:** Branca I. Pereira, Oliver P. Devine, Milica Vukmanovic-Stejic, Emma S. Chambers, Priya Subramanian, Neil Patel, Alex Virasami, Neil J. Sebire, Veronica Kinsler, Alexis Valdovinos, Claude Jourdan LeSaux, João F. Passos, Antony Antoniou, Malcom H. A. Rustin, Judith Campisi, Arne N. Akbar

**Affiliations:** 1 Division of Infection and Immunity, University College London, London, WC1E 6JF UK; 2 Institute of Histopathology, Great Ormond Street Hospital for Children, University College London, London, WC1N 3JH UK; 3 Paediatric Dermatology Department, Great Ormond Street Hospital for Children, University College London, London, WC1N 3JH UK; 4 Buck Institute for Research on Aging, 8001 Redwood Blvd, Novato, CA 94945 USA; 5 Institute for Cell and Molecular Biosciences & Newcastle University Institute for Ageing, Newcastle upon Tyne, NE1 7RU UK; 6 Department of Physiology and Biomedical Engineering, Mayo Clinic, Rochester, 55905 MN USA; 7 Department of Applied Sciences, Faculty of Health and Life Sciences, Northumbria University, Newcastle Upon Tyne, NE1 8ST UK; 8 Lawrence Berkeley National Laboratory, 1 Cyclotron Rd, Berkeley, CA 94720 USA

**Keywords:** Senescence, NK cells, CD8-positive T cells, Ageing

## Abstract

Senescent cells accumulate in human tissues during ageing and contribute to age-related pathologies. The mechanisms responsible for their accumulation are unclear. Here we show that senescent dermal fibroblasts express the non-classical MHC molecule HLA-E, which interacts with the inhibitory receptor NKG2A expressed by NK and highly differentiated CD8^+^ T cells to inhibit immune responses against senescent cells. HLA-E expression is induced by senescence-associated secretary phenotype-related pro-inflammatory cytokines, and is regulated by p38 MAP kinase signalling in vitro. Consistently, HLA-E expression is increased on senescent cells in human skin sections from old individuals, when compared with those from young, and in human melanocytic nevi relative to normal skin. Lastly, blocking the interaction between HLA-E and NKG2A boosts immune responses against senescent cells in vitro. We thus propose that increased HLA-E expression contributes to persistence of senescent cells in tissues, thereby suggesting a new strategy for eliminating senescent cells during ageing.

## Abstract (web summary)

Senescent cells increase with ageing and may cause inflammatory conditions, but how this accumulation is mediated is still unclear. Here the authors show that senescent cells express HLA-E to suppress NKG2A-mediated natural killer and CD8 T cell activation to avoid targeted elimination, while blocking NKG2A helps promote immunity against senescent cells.

## Introduction

Cellular senescence is an evolutionarily conserved mechanism with beneficial effects on tumour suppression^1^, wound healing^2^ and tissue regeneration^3^. During ageing, however, senescent cells accumulate in tissues and manifest deleterious effects, as they secrete numerous pro-inflammatory mediators as part of a senescence-associated secretory phenotype (SASP)^1,4^. The elimination of senescent cells in mouse models was shown sufficient to delay the onset or severity of several age-related phenotypes^5,6^. This has prompted the development of senolytic drugs that selectively target senescent cells^7^. Despite successful reversal of age-related pathologies in animal models, the use of senolytic drugs in humans may be hampered by their lack of specificity for senescent cells, leading to the risk of toxicity^7,8^. Therefore, alternative approaches that can be used in isolation or in combination with senolytic drugs to improve the elimination of senescent cells in humans should be explored.

Senescent cells can be recognised and eliminated by the immune system^9,10^. Different immune cell types including macrophages, neutrophils, natural killer (NK) cells and CD4^+^ T cells have been implicated in the surveillance of senescent cells, depending on the pathophysiological context^11–15^. Senescent cells become immunogenic by expressing stimulatory ligands like MICA/B that bind to NKG2D and activate their killing by NK cells^12,16^. Moreover, by secreting chemokines and cytokines, senescent cells can recruit immune cells into tissues that enable senescent cell clearance^13,17^. However, this secretory process may perpetuate a low-level chronic inflammatory state that underlies many age-related diseases^4,18^.

Despite the evidence for senescent cell clearance by the immune system, it is not yet clear why senescent cells accumulate during ageing and persist at sites of age-related pathologies^19^. A decline in immune function may contribute to incomplete elimination of senescent cells with age. Ageing has a great impact in both innate and adaptive immune systems, a process known as immunosenescence^20,21^. Alternatively, changes in major histocompatibility complex (MHC) expression can lead to escape from recognition by the immune system as previously described in cancer and virally infected cells in vivo^22–24^. Nevertheless, the effects of senescence on MHC expression are not fully understood.

Here, we show that senescent primary human dermal fibroblasts express increased levels of the non-classical MHC-class Ib molecule HLA-E. HLA-E inhibits immune responses against senescent cells by interacting with the inhibitory receptor NKG2A expressed on NK and highly differentiated CD8^+^ T cells. Accordingly, we find an increased frequency of HLA-E expressing senescent cells in the skin of old compared with young subjects. HLA-E expression is induced by SASP-related pro-inflammatory cytokines, in particular IL-6 and regulated by p38 signalling in vitro. Lastly, we show that that blocking HLA-E/NKG2A interactions in cell culture enhances NK and CD8^+^ T cell-mediated cytotoxicity against senescent cells. Taken together, these findings suggest that HLA-E expression contributes to the persistence of senescent cells in tissues. HLA-E may therefore represent a novel target for the therapeutic elimination of senescent cells in age-related diseases.

## Results

### Senescent cells express atypical MHC molecules

**Fig. 1:**
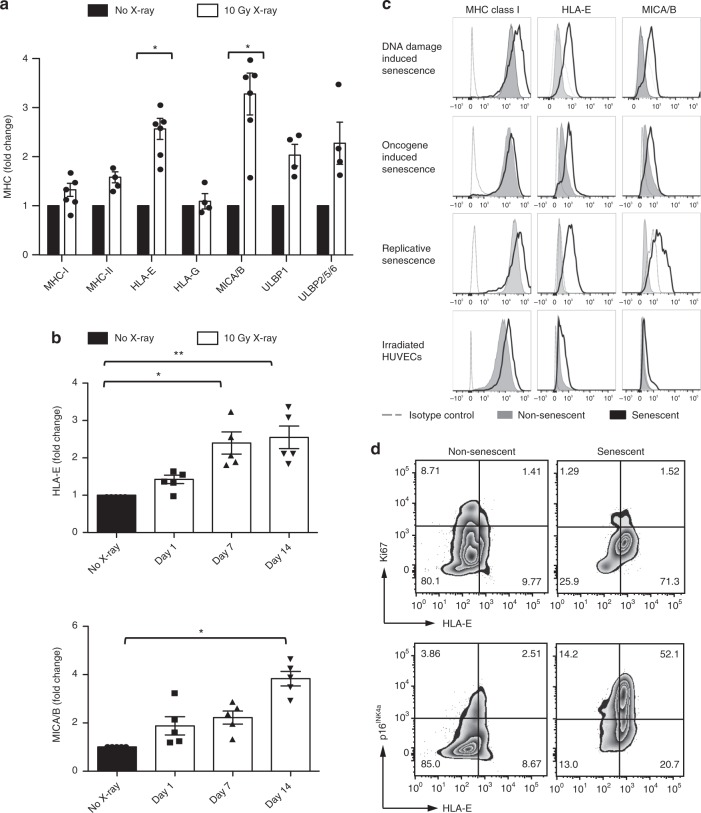
Senescent human fibroblasts express atypical MHC molecules. **a** Primary human fibroblasts were derived from the human skin and induced to senesce by ionising radiation (IR, 10 Gy X-ray). MHC expression by senescent fibroblasts (white bars) analysed at day 14 after IR using flow cytometry (*n* = 6 different donors for MHC-I, HLA-E and MICA/B, *n* = 4 for MHC-II, HLA-G and ULBP). Mean fluorescence intensity (MFI) values are shown as fold change compared with non-irradiated controls, set as one (black bars). **b** Time course of HLA-E and MICA/B expression at the indicated intervals after irradiation (*n* = 5). **c** Representative FACS plots of the total MHC-I, HLA-E and MICA/B expression in senescent fibroblasts induced by ionising radiation (DNA-damage induced senescence), *H-RAS* activation (oncogene-induced senescence) or continuous passaging (replicative senescence). MHC expression was compared between senescent (black lines), non-senescent (filled histograms) and isotype controls (dashed lines). Human umbilical vein endothelial cells (HUVECs) were irradiated (10 Gy), and MHC expression analysed by flow cytometry as previously described. **d** Flow-cytometry analysis of co-expression of HLA-E and Ki67 and p16^INK4a^ on irradiated fibroblasts (day 14 after irradiation) and non-irradiated controls. Numbers indicate percentages of cells per quadrant. The data are representative of at least three independent experiments from distinct samples. Statistical significance calculated with Mann–Whitney *U* test (**a**) and repeated measures ANOVA with Bonferroni correction (**b**). The data presented as means ± standard error of the mean (SEM). *p < 0.05, **p < 0.01, ***p < 0.001, ****p < 0.0001

Human fibroblasts were derived from skin explants of healthy volunteers and exposed to ionising radiation (X-ray) to induce senescence, as previously described^25–27^. We confirmed that a low dose of radiation (10 Gy) effectively arrested cell growth and induced the expression of senescence-associated markers, such as p16^INK4a^, persistent γH2AX foci and senescence-associated-β-galactosidase (SA-β-Gal) activity (Supplementary Fig. 1). We then examined the expression of MHC-class I, class II and non-classical MHC molecules (HLA-E, -F and -G) alongside MHC-related proteins of the MICA/B and ULBP families. We found that senescent cells have a significantly increased expression of atypical MHC molecules, such as MICA/B and HLA-E (Fig. 1a). A time course of cell surface expression of these ligands showed maximal expression between 7 and 14 days after irradiation (Fig. 1b), suggesting that MICA/B and HLA-E expression is associated with the establishment of senescence and is not only a result of acute DNA damage.

To assess whether this pattern of MHC expression was common to other forms of senescence, we monitored MHC expression in cells induced to senesce by oncogenic RAS activation (*H-RAS*^G12V^; oncogene-induced senescence; Supplementary Fig. 2A–C) or continuous passaging to replicative senescence (Supplementary Fig. 2D, E). Independently of the senescence-inducing stimuli, cells undergoing senescence consistently upregulated HLA-E and MICA/B (Fig. 1c). This expression was not unique to fibroblasts because human umbilical vein endothelial cells (HUVECs) exposed to a senescence-inducing dose of X-ray (Fig. 1c, bottom panels) also increased expression of HLA-E and MICA/B.

While the expression of MICA/B by senescent cells has been previously described^16,17^, the increased expression of HLA-E in senescent cells has not been shown previously. We used flow cytometry to confirm that HLA-E was expressed by senescent cells with additional markers. Non-irradiated fibroblasts expressed low levels of HLA-E and p16^INK4a^ (Fig. 1d, left panel). In contrast, the frequency of HLA-E-positive cells was significantly higher in irradiated cells frequently co-expressing p16^INK4a^ and rarely positive for Ki67 (Fig. 1d, right panel), indicating that the majority of HLA-E expressing cells were in cell-cycle arrest and expressed markers of senescence.

### HLA-E expression by senescent cells is regulated by p38

**Fig. 2:**
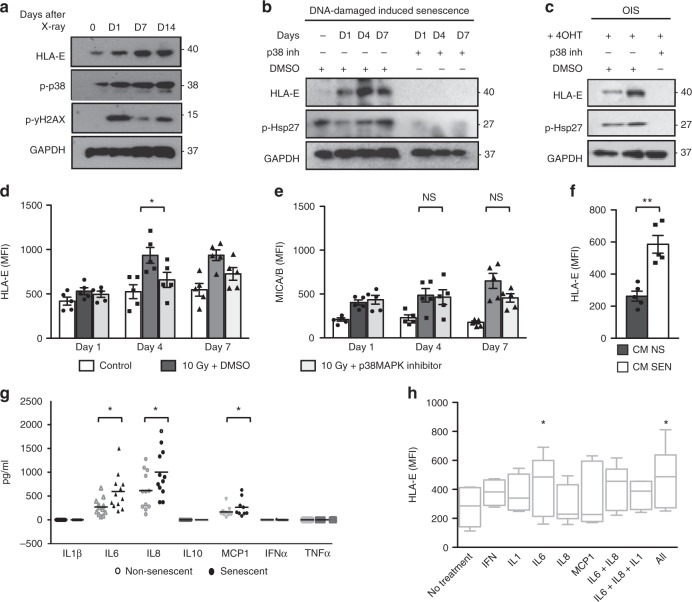
HLA-E expression on senescent cells is regulated by p38 and induced by IL-6. **a** Representative immunoblot of human fibroblasts at the indicated intervals after IR showing the kinetics of HLA-E, p38 (Thr180/Tyr182) and γH2AX (Ser139) phosphorylation. **b** Human fibroblasts were treated with p38 inhibitor BIRB796 (0.5 μM) or DMSO 12 h before, and for 7 days after IR. Immunoblot of HLA-E expression at the indicated intervals after IR compared with DMSO-treated controls. Undetectable levels of phospho-Hsp27 (p-Hsp27) confirm effective inhibition of p38. **c** IMR-90 ER:STOP/ER:RAS cells treated with 4-OHT to induce senescence (as demonstrated in Supplementary Fig. 2A–C) and treated with BIRB796 (0.5 μM) or DMSO over 7 days, followed by protein extraction and immunoblot analysis of HLA-E and p-Hsp27. GAPDH served as a loading control in **a**–**c**. Uncropped immunoblots are provided in the Source Data file. **d** The summary data of HLA-E and (**e**) MICA/B expression analysed by flow cytometry on irradiated fibroblasts treated with SB203580 (10 μM), compared with DMSO-treated and non-irradiated controls (*n* = 5). **f** Flow-cytometry analysis of HLA-E expression on fibroblasts exposed to the conditioned medium (CM) from senescent (SEN) or non-senescent (NS) cells for 48 h (*n* = 5). **g** Supernatant from irradiated senescent or early-passage fibroblasts analysed by cytokine-bead arrays to measure secreted cytokines (in pg/mL) (*n* = 12). **h** Fibroblasts were exposed to IL-6 (20 ng/mL), IL-8 (20 ng/mL), IL-1β (20 ng/mL) or IFNα (500 U/mL) or combinations of these for 48 h and analysed for HLA-E expression by flow cytometry (*n* = 6). The data are representative of at least three independent experiments from distinct samples. Comparison between groups performed with Kruskal–Wallis test in (**d**), (**e**) and Mann–Whitney *U* test in (**f**), (**g**) and (**h**). The data presented as means ± SEM. *p < 0.05, **p < 0.01, ***p < 0.001, ****p < 0.0001

To understand the molecular mechanisms regulating the expression of HLA-E on senescent cells, we investigated two major pathways that regulate senescence: the DNA-damage response (DDR) and p38 signalling. The DDR was previously implicated in increasing MICA/B expression in human and mouse fibroblasts exposed to genotoxic stress^28,29^. We therefore asked whether the DDR was also involved in regulating HLA-E expression after irradiation. We pretreated fibroblasts with the ATM inhibitor KU-55933 (10 μM) or DMSO 12 h before irradiation, and continuously thereafter over a period of 7 days. We monitored MHC expression by flow cytometry in non-irradiated cells and in vehicle- or KU-55933-treated cells 1, 4 and 7 days after irradiation. Consistent with previous reports, inhibition of ATM significantly decreased MICA/B expression after irradiation (Supplementary Fig. 3A). By contrast, ATM inhibition did not decrease the expression of HLA-E on irradiated fibroblasts (Supplementary Fig. 3B). Although we cannot exclude that HLA-E expression on senescent cells may be affected by the DNA-damage response, the increased expression of HLA-E at day 7 and day 14 after irradiation, when the expression of γH2AX is decreasing (Fig. 2a), suggests that the DNA-damage response is not necessary for HLA-E expression. By contrast, the similar kinetics of HLA-E and p38 phosphorylation at Thr180/Tyr182 (Fig. 2a) suggested a role of p38 signalling in regulating HLA-E expression on senescent cells. To test this, we pretreated senescent cells with the p38 inhibitor BIRB796^30,31^ or DMSO before irradiation and continuously thereafter for 7 days. As indicated by the levels of phosphorylated heat-shock protein 27 (p-Hsp27), a downstream target of p38, BIRB796 inhibited the activation of p38 after irradiation (Fig. 2b). Strikingly, the inhibitor also prevented the upregulation of HLA-E after irradiation, suggesting that activation of p38 is required for the upregulation of HLA-E expression after irradiation. These findings were confirmed in the oncogene-induced model of senescence (Fig. 2c) and also with an alternative p38 inhibitor SB203580, which inhibits p38 by a mechanism that differs from that used by BIRB796^30^ (Fig. 2d). Interestingly, we found no significant effect of p38 inhibition on MICA/B expression (Fig. 2e), suggesting that different pathways regulate the expression of MICA/B and HLA-E in senescent cells.

### HLA-E expression is induced by SASP cytokines

Since p38 is a key regulator of the SASP^32^, we asked whether SASP factors could increase HLA-E expression in a paracrine manner. We exposed early-passage fibroblasts to conditioned media (CM) from senescent or non-senescent cells. Non-senescent cells increased HLA-E expression within 48 h of exposure to CM from senescent cells (Fig. 2f).

We used cytometric-bead arrays to measure SASP factors secreted by senescent cells. We identified IL-6, IL-8 and MCP-1 as the most highly secreted SASP-related factors, as previously reported^32^ (Fig. 2g). Exposing non-senescent fibroblasts to these factors revealed a significant effect of IL-6, alone or in combination with other factors, in stimulating HLA-E expression (Fig. 2h). We cannot exclude, however, that other factors other than SASP-related pro-inflammatory cytokines may contribute to the upregulation of HLA-E in senescent cells.

### HLA-E ligates NKG2A and NKG2C on NK cells and CD8^+^ T cells

**Fig. 3:**
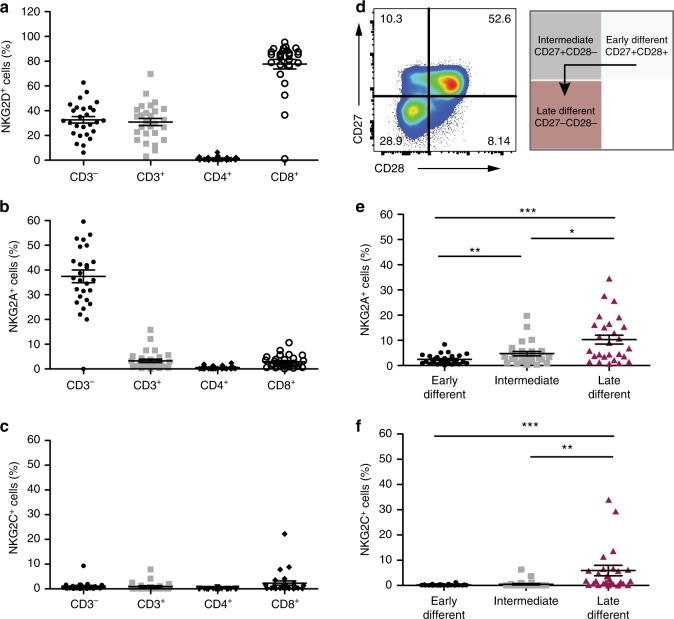
MICA/B and HLA-E bind NKG2 receptors expressed on NK and CD8^+^ T cells. Distribution of (**a**) NKG2D^+^, (**b**) NKG2A^+^ and (**c**) NKG2C^+^ cells on the indicated subsets of human lymphocytes from blood of healthy volunteers (*n* = 27; mean age 49.9; range, 27–83), analysed by flow cytometry. FACS sequential gating strategies represented in Supplementary Fig. 4. **d** Human CD8^+^ T cells were stratified according to CD27 and CD28 expression in early- (CD27^+^28^+^), intermediate- (CD27^+^28^−^) and late-differentiated (CD27^−^28) cells, as represented in the flow-cytometry plot. **e**, **f** The summary data of the distribution of NKG2A^+^ (**e**) and NKG2C^+^ (**f**) cells within early-, intermediate- and late-differentiated CD8^+^ T cells gated as in (**d**) in the same donors (*n* = 27; mean age 49.9; range, 27–83). Comparison between groups done with Friedman test with Dunn's correction for multiple comparisons in (**e**) and (**f**). The data presented as means ± SEM. *p < 0.05, **p < 0.01, ***p < 0.001, ****p < 0.0001

HLA-E and MICA/B bind receptors belonging to the Natural Killer Group 2 (NKG2) family, which regulate NK cell function^33^. MICA/B, and ULBP antigens, bind the stimulatory NK cell receptor NKG2D^29^, whereas HLA-E binds both the inhibitory NKG2A and stimulatory NKG2C receptors^34^. We analysed the distribution of NKG2 receptors on peripheral blood mononuclear cells (PBMCs) from healthy human volunteers (*n* = 27; mean age 49.9; range, 27–83 years) and found that NKG2D was highly expressed on NK (included in the CD3^−^ subset) and CD8^+^ T cells (Fig. 3a), as previously shown^29^. Expression of NKG2A and NKG2C, the cognate receptors for HLA-E, was not restricted to NK cells, and a proportion of CD8^+^ T cells also expressed these receptors (Fig. 3b, c). Human CD8^+^ T cells can be stratified by their relative expression of the co-stimulatory receptors CD28 and CD27, defining early (CD28^+^CD27^+^), intermediate (CD28^−^CD27^+^) and late (CD28^−^CD27^−^) stages of differentiation (Fig. 3d). The majority of NKG2A^+^ and NKG2C^+^ CD8^+^ T cells were highly differentiated (CD28^−^CD27^−^) cells (Fig. 3e, f), a subset that is expanded with age^35^. We found the proportion of NKG2A^+^ within the highly differentiated subset of CD8^+^ T cells to be significantly positively correlated with age (Supplementary Fig. 5A). This effect was not seen in the NKG2C^+^ compartment (Supplementary Fig. 5B). No association was found between the frequency of NKG2A^+^ cells and age within the CD3^−^ compartment (Supplementary Fig. 5C). Although we did not investigate the expression of NKG2A directly on NK cells in the CD3^−^ compartment, previous studies show that the expression of this receptor does not change on NK cells during ageing^36^, while others report an age-associated decrease in expression^37^.

### NK and CD8^+^ T cells target senescent fibroblasts via NKG2D

**Fig. 4:**
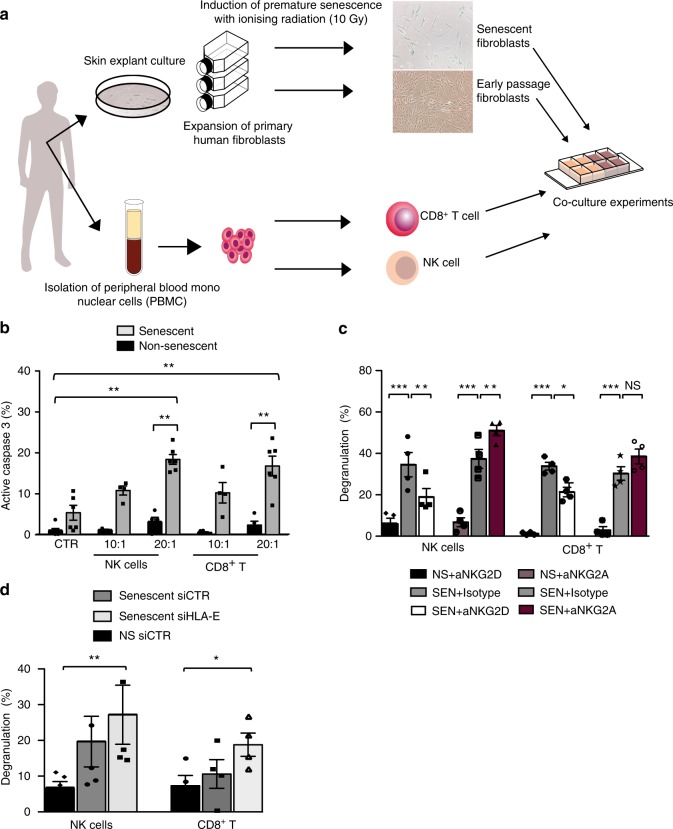
HLA-E/NKG2A blockade enhances senescent cell killing. **a** The experimental design of the autologous co-culture system to study immune surveillance of senescent cells: primary human fibroblasts from the skin of healthy volunteers were expanded and induced to senescence by ionising radiation. NK and CD8^+^ T cells from peripheral blood of the same donors (*n* = 5, age range 20–46) were used in co-culture experiments with autologous fibroblasts. **b** The summary data (*n* = 6) of active caspase 3 expression by non-senescent (black) and senescent fibroblasts (grey) after incubation with NK cells or CD8^+^ T cells, using different effector to target (E:T) ratios. Controls (CTR) indicate spontaneous activation of caspase 3 in fibroblasts cultured without effector cells. **c** NK and CD8^+^ T cells were pre-incubated with blocking antibodies to NKG2A (Z199), NKG2D (1D11) or isotype-matched controls, using an E:T ratio of 20:1. The summary data (*n* = 4) of degranulation of NK and CD8^+^ T cells towards senescent and non-senescent fibroblasts assessed by CD107a expression. **d** The cumulative data of CD107a expression in NK (*n* = 5) and CD8^+^ T cells (*n* = 4) after incubation with normal and senescent fibroblasts transfected with siRNA to HLA-E or a control siRNA. The data are presented as the index of degranulation (calculated as described in the Methods section) in **c** and **d**. Measurements were from distinct samples. Statistical analysis done with Mann–Whitney *U* test in (**b**) and one-way ANOVA with Bonferroni's multiple comparison test in **c** and **d**. The data presented as means ± SEM. *p < 0.05, **p < 0.01, ***p < 0.001, ****p < 0.0001

NK cells have been implicated in the surveillance and elimination of senescent cells through interaction between NKG2D and its ligands expressed on senescent cells^12,15–17^. Since CD8^+^ T lymphocytes also express the cognate receptors for MICA/B and HLA-E, we asked whether these cells could also participate in immune surveillance of senescent cells in analogy with NK cells. To avoid allogeneic reactions with T cells, we developed a co-culture system with skin-derived primary human fibroblasts and immune cells obtained from the same individuals (Fig. 4a). After inducing senescence with ionising radiation, we co-cultured senescent and non-senescent fibroblasts with freshly isolated autologous NK and CD8^+^ T cells.

Consistent with previous reports^11,12^, NK cells preferentially targeted senescent cells, as shown by significantly higher levels of active caspase 3 (measuring cell apoptosis) in senescent versus non-senescent fibroblasts when cultured with NK cells (Fig. 4b). Notably, CD8^+^ T cells could also induce significant activation of caspase 3 in senescent cells compared with non-senescent controls (Fig. 4b). These results were confirmed using a CD107a-based degranulation assay as a surrogate marker of cytotoxicity^38^, indicating that both NK and CD8^+^ T cells can target senescent fibroblasts in vitro (Fig. 4c). Pre-incubation of effector cells with NKG2D blocking antibody (1D11) decreased both NK and CD8^+^ T-cell degranulation towards senescent fibroblasts as compared with cells pretreated with a matched isotype control (Fig. 4c). By contrast, pre-incubation with NKG2A blocking antibody (Z199) significantly boosted the response of NK cells (Fig. 4c) and CD8^+^ T cells (Fig. 4c) against senescent cells, as compared with matched isotype controls. Collectively, these findings indicate that both NK and CD8^+^ T cells can kill senescent cells by an NKG2D-dependent mechanism, and that positive signals delivered by NKG2D ligation by MICA/B may be blocked if the inhibitory receptor NKG2A is engaged with its ligand HLA-E.

### NKG2A/HLA-E blockade enhances senescent cell surveillance

To directly investigate the role of HLA-E in inhibiting immune responses against senescent cells, we used RNA interference to inhibit HLA-E expression. At 36 h post transfection, the expression of HLA-E on senescent fibroblasts was decreased upon transfection with siRNA against HLA-E compared with the siRNA control (Supplementary Fig. 5D). This resulted in a significant increase in degranulation by autologous NK and CD8^+^ T cells (Fig. 4d), as compared with cells transfected with a scrambled siRNA control. These findings suggest that HLA-E expression on senescent cells decreases their susceptibility to elimination by NK and CD8^+^ T cells that express the inhibitory receptor NKG2A. The differences noted in the CD107a degranulation assay between antibody blocking experiments and experiments using siRNA transfection are most likely associated with the transfection protocol and incomplete silencing of HLA-E expression after transfection (Supplementary Fig. 5D).

### Relevance of HLA-E expression on senescent cells in vivo

**Fig. 5:**
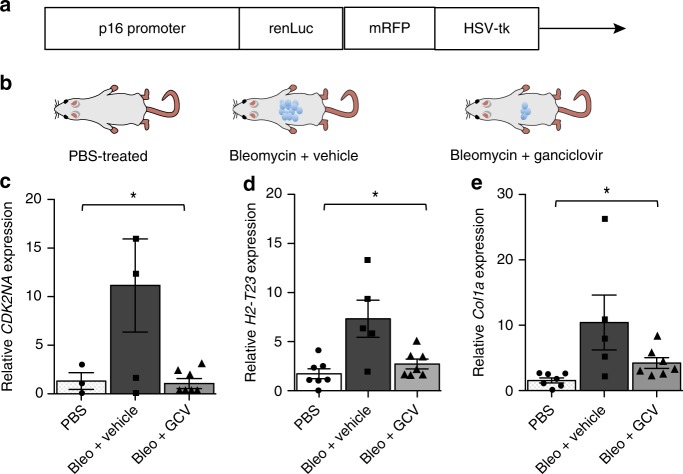
The expression of Qa-1^b^ (mouse homolog of HLA-E) in p16-3MR mice. **a** Schematic of the p16-3MR (trimodality reporter) fusion protein, containing functional domains of a synthetic Renilla luciferase (LUC), monomeric red fluorescent protein (mRFP) and truncated herpes simplex virus 1 (HSV-1) thymidine kinase (HSV-TK) driven by the p16 promoter. **b** p16-3MR mice were treated with bleomycin (intra-tracheal injection, 1.9 UI/Kg), ganciclovir (GCV, 25 mg/kg; daily i.p. injections) or PBS; **c**–**e** qRT-PCR was used to quantify levels of mRNAs encoding p16^*INK4a*^ (*CDKN2A*), Qa-1 (*H2-T23*) and collagen (*COL1A1*) in lungs from mice treated with PBS (white bar), bleomycin + vehicle control (black bars) and bleomycin + GCV (grey bars). mRNA levels for the indicated genes were normalised to tubulin and presented as fold difference relative to PBS-treated mice. Statistical analysis between groups performed with one-way ANOVA with Bonferroni's multiple comparison test. The data presented as means ± SEM. *p < 0.05, **p < 0.01, ***p < 0.001, ****p < 0.0001

To determine whether HLA-E expression was also elevated on murine senescent cells in vivo, we used p16-3MR reporter mice model that allows the identification of p16^INK4a^-positive (senescent) cells via the p16^INK4a^ promoter driven expression of luciferase (Fig. 5a). Importantly, it also enables the selective elimination of senescent cells by administration of ganciclovir (GCV) through p16^INK4a^ promoter driven expression of the herpes simplex virus 1 (HSV-1) thymidine kinase (HSV-TK)^2^. We treated p16-3MR mice with the genotoxic drug bleomycin to induce senescence and fibrosis in the lung^39^, with or without ganciclovir to eliminate senescent cells (Fig. 5b). We harvested whole lungs and analysed them by quantitative polymerase chain reaction for genes encoding Qa-1^b^ (*H2-T23*, the mouse homologue of HLA-E), p16^INK4a^ (*CDKN2A*) and collagen (*COL1A1*) as a proxy marker of fibrosis. *CDKN2A* mRNA levels increased 14 days after treatment with bleomycin (Fig. 5c), as did *H2-T23* mRNA levels (Fig. 5d). Furthermore, when mice were treated with GCV to eliminate p16^Ink4a^-positive cells, *H2-T23* gene expression declined to control levels (Fig. 5d). Likewise, *COL1A1* mRNA levels increased upon induction of senescence by bleomycin and declined after eliminating senescent cells with GCV. These results suggest that fibrosis is associated with the development of senescence and is alleviated when senescent cells are cleared (Fig. 5e).

**Fig. 6:**
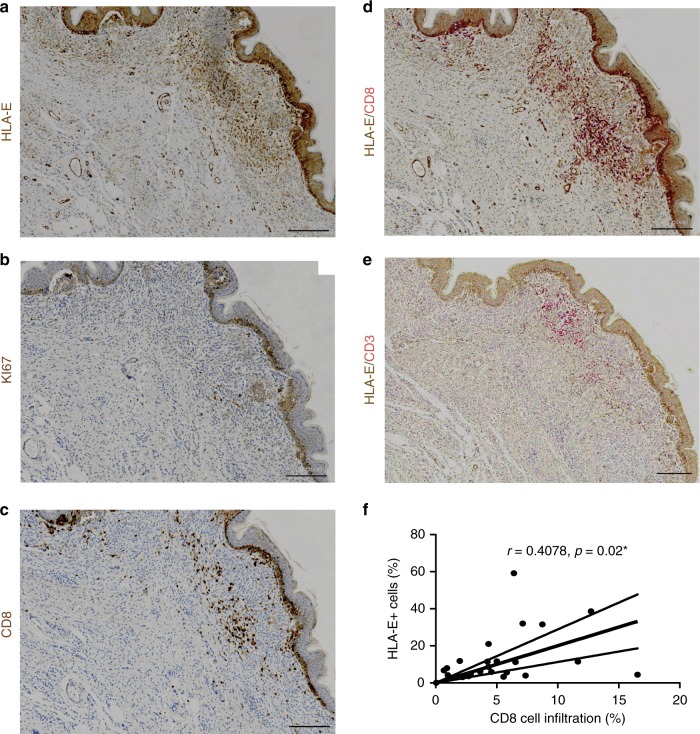
HLA-E expression by senescent cells in human melanocytic nevi. Formalin-fixed paraffin-embedded tissue arrays of human melanocytic nevi were analysed by immunohistochemistry using (**a**) HLA-E (MEM-E/02), (**b**) Ki67, (**c**) CD8 specific antibodies. **d** Double-staining with antibodies for HLA-E (brown) and CD8 (red) showing CD8^+^ infiltrates surrounding areas with strong HLA-E expression. **e** Double-staining with HLA-E (brown) and CD3 (red) showing that part of the CD8^+^ infiltrates are also positive for CD3, identifying them as CD8^+^ T cells. Scale bar = 50 μm. **f** Correlation between the frequency of HLA-E^+^ cells and CD8^+^ infiltrates in human melanocytic nevi (*n* = 24) assessed by Spearman test

To assess the biological relevance of HLA-E expression by senescent cells in humans, we studied congenital melanocytic nevi (Fig. 6). Previous studies showed that melanocytic nevi are enriched with cells expressing markers of senescence and thus are likely an example of oncogene-induced senescence in vivo^40,41^. We immunostained formalin-fixed, paraffin-embedded (FFPE) tissue arrays of congenital and acquired human melanocytic nevi for HLA-E expression (Fig. 6a). We analysed the staining using Image Analysis Software and scored HLA-E expression based on the percentage of HLA-E^+^ cells, ranking as negative (score 0: between 0 and 1%), mild (score 1: 1–5%), moderate (score 2: 5–10%) or intense (score 3: >10%). In each array, normal skin and melanoma samples were used as controls (Supplementary Fig. 6). Among 24 lesions studied, HLA-E expression was detectable in most (23/24) human melanocytic nevi. In 10/24 (41.7%) lesions, we observed strong positivity for HLA-E (score 3), with frequencies of HLA-E positive cells up to 30% (Supplementary Table 1). Sections were also stained for Ki67 as a marker of proliferation, which demonstrated that cells expressing HLA-E were rarely Ki67 positive (Fig. 6b). To determine levels of immune cell infiltration, the same tissue arrays were stained for CD8 (Fig. 6c) and the percentage of these cells was calculated using the same software (mean 5.29 ± 4.01%, range 0.05–16.5%). Co-staining for CD8 and HLA-E showed that CD8^+^ cells frequently co-localised with HLA-E^+^ cells (Fig. 6d). As NK cells can also stain positively for CD8, tissue arrays were co-stained with CD3 to distinguish NK from T cells. This analysis confirmed the presence of CD3^+^ CD8^+^ T lymphocytes, however, these cells did not account for all the immune cell infiltration (Fig. 6e). We conclude that both T cells and NK cells associate with HLA-E expressing cells in melanocytic nevi, and we observed a significant positive correlation between the frequency of HLA-E^+^ cells in melanocytic nevi and the percentage of CD8^+^ T-cell infiltration in melanocytic nevi (Fig. 6f). Therefore, HLA-E expression is common in human melanocytic nevi and may explain why these nevi persist for decades in tissues, despite the presence of immune cell infiltrates.

### HLA-E expression by senescent fibroblasts in human skin

**Fig. 7:**
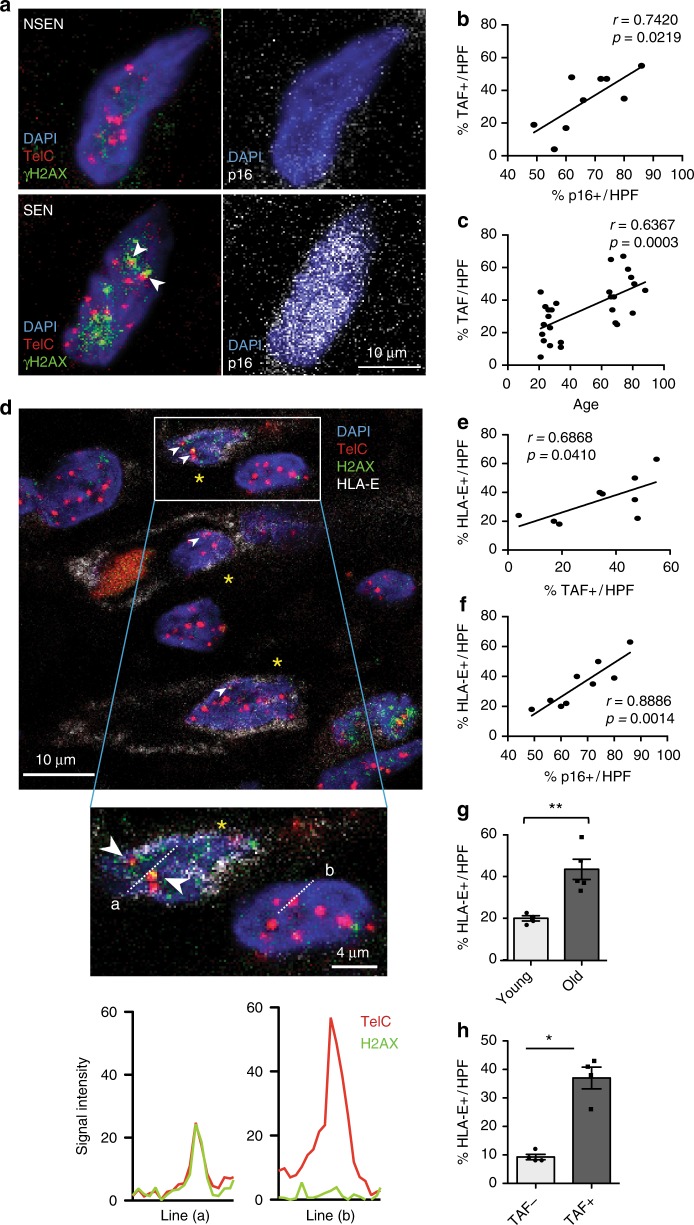
HLA-E expression by senescent fibroblasts in human skin during ageing. **a** Histological sections from healthy donors were stained for DAPI (blue), TelC (red punctate intranuclear), γH2AX S139 (green) and p16^INK4A^ (white). Telomere-associated γH2AX foci (TAF) are shown (white arrow heads) in non-senescent (NSEN; top panels) and senescent (SEN; bottom panels) cells in the dermis of the human skin. Original image shown in Supplementary Fig. 7E and provided in the Source Data file. Scatterplot showing the relationship between the frequency of p16^INK4A+^ cells (**b**) and donor age (**c**) with the frequency of TAF^+^ cells in the interstitial dermis of the human skin. **d** Skin sections were stained for DAPI (blue), TelC (red punctate intranuclear), γH2AX S139 (green) and HLA-E (white). Telomere-associated γH2AX foci (TAF) are shown (white arrow heads) in senescent HLA-E^+^ cells (yellow asterisks) of the human dermis. The signal intensity of TelC and γH2AX along lines (a) and (b) are represented in histogram format and both signals overlap in the senescent, but not the non-senescent cell. **e** Correlation of HLA-E^+^ cells and TAF^+^ cells in the interstitial dermis of human skin. **f** Correlation of HLA-E^+^ cells and p16^INK4A+^ cells in the interstitial dermis of the human skin. **g** The frequency of HLA-E^+^ cells present in the superficial dermis of young (*n* = 4) and old (*n* = 5) human skin. **h** The frequency of HLA-E^+^ cells in the TAF^+^ and TAF^−^ populations in the dermis of young (*n* = 4) and old (*n* = 5) human skin. The data are represented as mean ± SEM. Statistical significance calculated with Mann–Whitney *U* test. *p < 0.05, **p < 0.01, ***p < 0.001, ****p < 0.0001

Senescent cells have been shown to increase in many tissues during ageing, including in the skin^42^. To investigate whether senescent cells express HLA-E in healthy individuals in vivo, we analysed histological sections of normal human skin from young (<40 years) and old (>65 years) healthy donors using confocal microscopy. Firstly, we identified non-senescent (Fig. 7a, top panels) and senescent cells (Fig. 7a, bottom panels) by staining for telomere-associated DNA-damage foci (TAF; left hand panels) or p16^INK4A^ (Fig. 7a, right panels). In sections that were stained for both TAF and p16^INK4A^, there was a significant correlation between both senescent markers (Fig. 7b). We subsequently used TAF staining alone to identify senescent cells. There was a highly significant correlation between senescent (TAF^+^) cells in the interstitial dermis and increasing donor age (Fig. 7c).

As seen in representative confocal micrographs of interstitial dermal cells of the human skin (Fig. 7d), TAF^+^ cells co-stain for HLA-E confirming that HLA-E expressing cells display markers of senescence. There was a positive correlation between the frequencies of HLA-E^+^ and TAF^+^ cells (Fig. 7e) and HLA-E^+^ and p16^INK4A+^ cells in the same skin sections (Fig. 7f). Finally, we found a significant increase in the frequency of HLA-E^+^ cells in older donors as compared with young (Fig. 7g). A significantly higher proportion of senescent (TAF^+^) cells expressed HLA-E^+^ compared with non-senescent (TAF^−^) cells (Fig. 7h). The majority of TAF^+^ senescent cells were dermal fibroblasts, a subset of which we identified as staining positive for the fibroblast-specific protein-1 (FSP1)^43^ (Supplementary Fig. 7A). A similar proportion of FSP1^+^ cells were found in the interstitial dermis of young and old subjects (Supplementary Fig. 7B). There was a significantly increased frequency of TAF^+^ senescent cells in both FSP1^+^ and FSP1^−^ populations in the skin of old donors compared with young (Supplementary Fig. 7C, D), indicating that other cells as well as fibroblasts contribute to the senescent cell population in older individuals.

## Discussion

This study identifies a mechanism of immune evasion that may contribute to the persistence of senescent cells in tissues. We found that fibroblast senescence is associated with an upregulation of HLA-E, which has restricted expression in non-senescent cells. The increased HLA-E expression on senescent fibroblasts impairs their elimination by NK and CD8^+^ T-cells expressing the inhibitory receptor NKG2A. Previous transcriptomic studies of replicative senescent human fibroblasts included HLA-E in the list of genes that increased more than twofold compared with proliferative cells, corroborating our findings^44^. Functionally, we show that both NK and CD8^+^ T lymphocytes can target senescent cells through an NKG2D-dependent mechanism. Despite expressing high levels of NKG2D, the simultaneous presence of the inhibitory receptor NKG2A in NK and CD8^+^ T cells may compromise the immune-mediated clearance of senescent cells. Even if the activating receptor NKG2C is co-expressed on the same effector cell, the affinity of HLA-E binding to NKG2C is lower compared with NKG2A and inhibitory signals dominate and override activation signals^45^. The expansion of NKG2A^+^ CD8^+^ T cells with age may explain why the immune system is less effective at eliminating senescent cells in old subjects. Likewise, the composition and function of NK cells is highly susceptible to ageing^20,36^; however, we did not find significant age-related differences in the frequency of NKG2A^+^ cells within the CD3^−^ compartment in line with previous studies^36,46^, whereas other studies suggest that the frequency of NKG2A^+^ NK cells may decrease with age^37^. Despite this, the relatively high expression of NKG2A receptors on NK cells (Fig. 3b) is likely to have important implications in the surveillance of senescent cells. This is supported by the more pronounced effect of NKG2A inhibition in NK compared with CD8^+^ T cells (Fig. 4c). Because the responses to NKG2D and NKG2A blockade were only partial, we cannot exclude that additional receptors on NK and CD8^+^ T cells may be important in this interplay, as suggested previously^15^.

NK cells and their crosstalk with other immune cells have been shown to be major mediators in the clearance of senescent cells in the context of liver fibrosis^12,16^, hepatocellular carcinoma^17^ or multiple myeloma^15^. This suggests that the mechanism of senescent cell persistence through HLA-E expression leading to inhibition of T and NK-cell killing may also be relevant in these contexts. However, it may not be applicable to other pathophysiological contexts, where the elimination of senescent cells seems to be dependent on other immune cells in particular monocytes/macrophages^13,14^. One situation where decreased senescent cell clearance may be important is in the persistence of melanocytic nevi that contain senescent cells that co-localise with immune cells. The expression of HLA-E at these sites would explain why these cells are not cleared.

Mechanistically, we demonstrate that HLA-E expression on senescent cells is regulated by p38 signalling in vitro. Previous studies showed that pro-inflammatory cytokines such as IL-1β, TNF and IFN-γ can induce HLA-E expression on endothelial cells^47^. We found that the SASP-related cytokine IL-6 induced HLA-E expression on non-senescent cells in a paracrine fashion. IL-6 has been implicated in many of the SASP-induced effects on surrounding cells, including epithelial-to-mesenchymal transitions, tumour progression and fibrinogenesis^48^. Moreover, increased levels of IL-6 and IL-8 are found in the sera of elderly patients, and contribute to the systemic low-level chronic inflammation that has been linked to many age-related diseases^18^. The upregulation of HLA-E expression by IL-6 thus suggests that sustained inflammation may also contribute to the persistence of senescent cells in tissues, further contributing to the pathogenesis of age-related diseases.

We cannot exclude that other mechanisms may contribute to the overexpression of HLA-E on senescent cells. It would be interesting to investigate whether factors other than SASP-related pro-inflammatory cytokines such as reactive oxygen species may contribute to the upregulation of HLA-E in senescent cells. While HLA-E mRNA can be found in almost all nucleated cells, the stability of HLA-E expression at the cell surface depends on the availability of highly conserved nonameric peptides commonly generated after trimming of the leader sequence of classical MHC-class I molecules^49^. There is accumulating evidence that HLA-E can present a diverse repertoire of peptides, derived from viral particles, heat-shock proteins^50^ and other stress-induced peptides^51^. Recent studies indicate that CD8^+^ T cells can also recognise foreign peptides presented by HLA-E via the TCR eliciting CD8^+^ T cell-specific immune responses^52^. Further investigation is required to determine whether HLA-E can present senescent-specific antigens in this context.

Collectively, our findings support a model for immune-mediated senescent cell persistence where a balance between activating and inhibitory signals will determine the outcome of the NK and T-cell immune response to senescent cells. From a therapeutic perspective, interventions blocking the interaction between HLA-E and the inhibitory receptor NKG2A may represent promising strategies to improve the immune clearance of senescent cells. Humanised monoclonal antibodies targeting NKG2A (Monalizumab) are readily available and currently in clinical trials for cancer^53^. It will be important to determine how the combination of immune modulators such as anti-NKG2A, senolytic drugs as well as direct inhibitors of the SASP can be used for the elimination of senescent cells in vivo^54,55^.

## Methods

### Blood and skin sample collection

Blood and skin samples were obtained from healthy research volunteers. The study was approved by the Research Ethics Committee of University College London (06/Q0502/92) and the National Health Service Research Ethics Service (11/LO/1846). All volunteers provided written, informed consent. In total, 5 -mm skin punch biopsies were obtained from sun-protected areas of the forearm of healthy young subjects (<40 years of age) and old subjects (>65 years of age). Biopsies were either snap-frozen in liquid nitrogen for later histological analysis or prepared for skin-explant culture to derive primary fibroblasts as described^25^. Peripheral blood mononuclear cells (PBMC) were isolated by density-gradient centrifugation (Ficoll–Hypaque, Amersham) from blood of healthy donors. Untouched NK and CD8^+^ T cells were freshly isolated by magnetic activated cell sorting (MACS, Miltenyi Biotec) using a negative selection procedure (>95% purity).

### Induction of senescence

Primary human fibroblasts were induced to senesce by exposure to X-ray radiation at a total dose of 10 Gy at a rate of 5 Gy/min. Oncogene-induced senescence was induced in IMR-90 human diploid fibroblasts transduced with a vector expressing *H-RAS*^G12V^ (ER:RAS) or a control insert (STOP), both containing a tamoxifen-inducible oestrogen receptor fusion protein (ER:STOP). Cells were treated with 4-hydroxytamoxifen (4-OHT) (Sigma-Aldrich) at 200 nM to activate *H-RAS*^G12V^, as described^56^. Replicative senescence was induced by continuous passaging of fibroblasts until they reached a plateau in their growth curve. Cumulative population doublings (PD) were calculated using the following equation:

1 $${\mathrm{PD}} = \frac{{\log \;n_{\mathrm{c}} - {\mathrm{log}}\;n_{\mathrm{s}}}}{{{\mathrm{log}}\;2}}$$

where *n*_c_ represents the number of cells counted after expansion and *n*_s_ represents the number of cells seeded.

### Senescence-associated β-galactosidase staining

Cells were fixed and stained using the Senescence-Associated β-Galactosidase Staining Kit (Cell Signaling, 9860), following the manufacturer's instructions. After staining, cells were incubated for 12–16 h at 37 °C, then visualised by phase-contrast microscopy.

### Telomere fluorescence in situ hybridisation

Six micrometers cryosections were obtained from skin biopsies and prepared on poly-L-lysine coated glass slides (Cytospin, Thermo Scientific). Staining for telomere-associated ɣH2AX foci (TAF) was then performed. Sections were stained for ɣH2AX (Ser139, Cell Signaling #9718, 1:250), p16^INK4a^ (Abcam, ab108349, 1:100 or Sigma SAB5300499, 1:100) and HLA-E (clone 3D12, eBioscience, 1:100), followed by incubation with secondary antibody conjugated to various fluorochromes and washed in formamide/SSC prior to mounting with Vectorshield/DAPI (Vector Laboratories). Slides were air dried prior to hybridisation for 2 h with 40 pM PNA probe targeting the TelC telomeric repeat (Panagene, TelC Cy3, #14 1224PL-01). Imaging of TAF foci was then performed using a Leica SPE2 confocal microscope (Leica Microsystem). Imaging consisted of obtaining Z-stacks with a step-size of 0.5 µm. Analysis was performed using Fiji image analysis software (Fiji.sc).

### Western blotting

Cells were harvested by trypsinization, washed in PBS and lysed with radio-immunoprecipitation assay (RIPA) buffer (Sigma-Aldrich) supplemented with protease and phosphatase inhibitors (GE Healthcare) for 30 min on ice. Protein concentrations were determined using the Pierce BCA Protein Assay Kit (Thermo Scientific). Cell lysates (10 or 20 μg of the total protein) were diluted in SDS sample buffer with reducing agent (NuPage, Life Tecnologies) and boiled for 5 min at 95 °C. Cell lysates were separated by protein electrophoresis at 120 V for 2 h using 10% Bis-Tris pre-cast gels (NuPage) and transferred overnight at 4 °C onto Hybond-P PVDF membranes (GE Healthcare). After blocking, membranes were probed with primary antibodies overnight at 4 °C, washed and incubated with HRP-conjugated secondary antibodies (GE Healthcare, 1:4000) for 1 h at room temperature. Antibodies were detected using the ECL detection kit (GE Healthcare). Prior to re-probing with different antibodies, membranes were stripped at 37 °C in agitation using Restore stripping buffer (Thermo Scientific). Protein bands were quantified using ImageJ software. The integrated density of each band was measured using the gel analysis function of ImageJ, normalised to GAPDH. Primary antibodies were rabbit polyclonal anti-histone γH2A.X (pS139), anti-Hsp27 (pS78), anti-p38 MAPK (pThr180/Tyr182), anti-p53 and anti–GAPDH (all from Cell Signaling). For HLA-E expression, we used mouse monoclonal anti-HLA-E (MEM-E/02, Santa Cruz Biotechnology), as described^57^. All primary antibodies were used at a dilution of 1:1000. Uncropped immunoblots are provided in the Source Data file.

### Flow cytometry

For fibroblast staining, cells were washed in PBS and harvested after trypsin treatment, centrifuged at 1200 rpm for 10 min and resuspended in ice-cold PBS. Flow-cytometric analysis of surface expression of MHC molecules was performed after a 30-min incubation at 4 °C in the presence of saturating concentrations of antibodies (Supplementary Table 2) and a live/dead stain. Proliferation was assessed by staining for Ki67 (mouse anti-human Ki-67 set, BD Biosciences), p16^Ink4a^ expression was performed using PE Mouse anti-Human p16 set (BD Biosciences, 556561). For the detection of γH2AX (Ser139), we used phosphoflow cytometry after fixation (10 min at 37 °C with Cytofix Buffer), permeabilisation (30 min at 4 °C with ice-cold Perm Buffer III), washing (twice with Stain Buffer, all from BD Biosciences) and incubation for 30 min at room temperature with Alexa Fluor 488-conjugated antibody to γH2AX (Ser139) (clone 2F3; BioLegend). Multi-parametric flow cytometry was used for phenotypic analysis of surface expression of NK-cell receptors (antibodies listed in Supplementary Table 2). Samples were acquired on a LSR II flow cytometer (BD Biosciences) and analysed using FlowJo software (TreeStar).

### Autologous co-culture system

Paired blood and skin samples were obtained from healthy donors (*n* = 5, age range 20–46). Skin-derived primary human fibroblasts were irradiated and allowed to recover in culture until they developed a senescent phenotype. Senescent fibroblasts (14–21 days after irradiation) were seeded onto flat-bottom 48-well plates (20 × 10^4^ cells per well) and grown to confluence at 37 °C and 5% CO_2_. Early-passage fibroblasts (passages 3–9) were used as non-senescent controls. When confluent, monolayers were washed and incubated with IL-2 activated NK and CD8^+^ T cells freshly isolated from the same individuals.

### Cytotoxicity assays

Cytotoxicity was assessed after 6-h incubation by quantifying active caspase 3 levels in target cells using flow cytometry (Caspase 3 Assay kit, 556485, BD Biosciences). Baseline expression of caspase 3 in fibroblasts incubated with medium only was used as a negative control. Fibroblasts treated with camptothecin (8 μg/mL; Sigma-Aldrich) were used as positive controls. Alternatively, CD107a (lysosomal-associated membrane protein-1, LAMP-1) expression was used as a marker of CD8^+^ T and NK cells degranulation, as described^38^. The data are presented as an index calculated as (sample degranulation–spontaneous degranulation)/(maximum degranulation−spontaneous degranulation) × 100.

### Antibody blockade experiments

Freshly purified NK and CD8^+^ T cells were pre-incubated with 1 μg/mL anti-NKG2D (1D11, eBioscience), 1 μg/mL anti-NKG2A (Z199, Beckman Coulter) or respective isotype controls followed by incubation with senescent or non-senescent fibroblasts.

### Transfection of cells with small interfering RNAs

Primary human fibroblasts were transfected with siRNAs for HLA-E (sc-62470, Santa Cruz Biotechnology) or a control siRNA (sc-37007, Santa Cruz Biotechnology) using the Amaxa Human Dermal Fibroblast Nucleofector Kit (Lonza) as per the manufacturer's protocol. HLA-E siRNA (h) is a pool of three siRNAs that inhibit HLA-E expression. Transfection efficiency was confirmed by measuring protein content by flow cytometry, typically 36–48 h after transfection.

### Treatment with inhibitors in vitro

Cells were treated with 10 μM KU-55933 (EMD Millipore) or 10 μM CGK733 (Calbiochem). Cells were treated with 0.5 μM BIRB796 (Selleck Chemicals) or 10 μM SB203580 (Calbiochem) for 12 h before and continuously after irradiation over 7 days, with daily replenishment with fresh media and inhibitors. A solution of 0.1% DMSO in the DMEM was used as a vehicle control.

### Cytometric bead array

Conditioned media from senescent and early-passage fibroblasts were collected and assessed for cytokine production according to the manufacturer's protocol. Samples were analysed in a BD Verse flow cytometer (BD Biosciences). The lower limit of detection for each analyte was 1.5 pg/mL.

### Treatment with conditioned medium and cytokines

Conditioned media (CM) were generated by culturing cells in the serum-free DMEM supplemented with penicillin/streptomycin for 24 h. CM were normalised to the cell number and clarified by centrifugation and filtration and diluted 1:1 with 10% FBS–DMEM. Early-passage fibroblasts were incubated with diluted CM for 48 h before analysis for HLA-E expression by flow cytometry. Recombinant human cytokines IL-6 (20 ng/mL), IL-8 (20 ng/mL), IL-1β (20 ng/mL) or IFNα (500 U/mL, all from Peprotech UK) were diluted in complete DMEM.

### Tissue arrays of congenital melanocytic nevi

Tissue arrays of congenital melanocytic nevi (CMN) were obtained from the Paediatric Dermatology Department at the Great Ormond Street Hospital. Samples in these tissue arrays were taken as part of clinical care, and informed consent was obtained from all patients, after approval by the Research Ethics Committee of Great Ormond Street Hospital and the University College London Institute of Child Health. Each array included 14–16 formalin-fixed paraffin-embedded sections of CMN from different patients and normal skin, acquired melanocytic nevus and malignant melanoma as controls.

### Immunohistochemical staining

Immunohistochemical staining of tissue arrays of congenital melanocytic nevi was performed on the automated Leica BOND-MAX immunostainer (Leica Biosystems) following deparaffinization and heat-induced epitope retrieval (with Leica HIER solution ER2 at pH9). Staining was detected using Leica Bond polymers. Dual staining of HLA-E and CD8/CD3 was performed in the same tissue section using a sequential staining technique and the Chromoplex Dual Staining Detection system (Leica Biosystems). All antibodies were optimised for use on paraffin sections with appropriate positive and negative controls.

### Image analysis

Tissue array slides were scanned on a LEICA SCN400F digital slide scanner (Leica Microsystems), and images were analysed on the SlidePath Digital Image Hub (Leica) with Definiens Tissue Studio 3.6 (Definiens AG). After manually selecting regions of interest (ROI) in each section, analysis algorithms were as follows: the software determined the area of each ROI (in µm²) and identified and counted the number of nuclei (# blue), the number of HLA-E^+^ cells (# brown), the number of CD8^+^ cells (# red) and the number of CD8^+^ cells bordering HLA-E^+^ cells (# red bordering brown) per ROI. Counts were expressed as cells/µm². Using this information, we defined a function for HLA-E intensity ((# brown/# blue)*100), CD8 infiltration ((# red/# blue)*100) and co-localisation of CD8 and HLA-E ((# red bordering brown/# red)*100). HLA-E intensity and CD8 infiltration were defined according to the frequency of HLA-E^+^ and CD8^+^ cells and scored as: negative (score 0: between 0 and 1%), mild (score 1: 1–5%), moderate (score 2: 5–10%) and intense (score 3: >10%). The complete data analysis is available in Supplementary Table 3.

### The p16-3MR mouse model

Experiments with p16-3MR mice were performed under protocols approved by the Buck Institute's Animal Care and Use Committee. Mice were given bleomycin (1.9 U/Kg, daily) or PBS via intratracheal instillation for 14 days. Ganciclovir (25 mg/kg; daily) or vehicle were administered via intra-peritoneal injection for 7 days starting 1-week after bleomycin treatment. Animals were euthanized and tissues collected for analysis.

### RNA isolation and real-time quantitative PCR

The total RNA from mouse lung was isolated using Directzol RNA miniprep (Zymo Research), following the manufacture’s protocol. RNA was reverse-transcribed using TaqMan Universal PCR Master Mix (Applied Biosystems), and cDNA was analysed by real-time qPCR using the Roche Universal Probe Library (UPL) system. All reactions were performed in triplicate, and mRNA levels were normalised to averages of actin and tubulin, unless noted otherwise. The relative amount of mRNA was determined using the comparative threshold (Ct) method by normalising target cDNA Ct values to that of actin/tubulin. Fold changes were calculated relative to control (PBS) for each group using the formula 2*e* − ΔΔCt.

Primer sequences and probes used:

Mouse actin: F 5′-CTAAGGCCAACCGTGAAAAG-3′, R 5′-ACCAGAGGCATACAGGGACA-3′, UPL Probe #64;

Mouse tubulin: F 5′-CTGGAACCCACGGTCATC-3′, R 5′-GTGGCCACGAGCATAGTTATT-3′, UPL Probe #88;

Mouse *H2-T23*: F 5´-AGCCCCTCACCCTGAGAT-3′, R 5′-ACCACAGCTCCAAGGATGAT-3′, UPL Probe #94;

Mouse *CDKN2A*: F 5′-AATCTCCGCGAGGAAAGC-3′, R 5′-GTCTGCAGCGGACTCCAT-3′, #91.

### Statistical analysis

Statistical analysis was performed using Prism (GraphPad Software). Depending on normality of the data, comparisons were performed using the Student *t* test, the non-parametric Mann–Whitney U test (for two groups), the Wilcoxon signed rank test (for >2 paired groups), Kruskal–Wallis (for >2 unpaired groups) or Friedman (for >2 paired groups) one-way ANOVA tests, as appropriate. Linear regression analysis was performed to generate lines of best fit, and correlations between variables were analysed using Pearson's or Spearman’s rank correlation coefficients (r). Two-tail *P-*values were calculated and considered significant if *P* < 0.05. The data are presented as means ± standard error of the mean (SEM) unless otherwise stated. *p < 0.05, **p < 0.01, ***p < 0.001, ****p < 0.0001.

### Reporting summary

Further information on research design is available in the Nature Research Reporting Summary linked to this article.

## Supplementary information

Supplementary Information

Reporting Summary

Source Data

## Data Availability

The source data underlying the figures in the manuscript are provided as a Source Data file. All other data are included in the supplemental information or available from the authors upon reasonable requests.
