# Supplementary material for: Senescent cells evade immune clearance via HLA-E-mediated NK and CD8+ T cell inhibition
Source: Nat Commun. 2019 Jun 3;10:2387. doi: 10.1038/s41467-019-10335-5 (PMC6547655; doi:10.1038/s41467-019-10335-5)
Supplement: Supplementary file 1 — Supplementary Information [file 41467_2019_10335_MOESM1_ESM.pdf]

Supplementary Information

Senescent cells can evade immune clearance by expressing HLA-E that inhibits immune responses mediated by NK and $CD8^+$ T cells

Pereira *et al*

Supplementary Figures

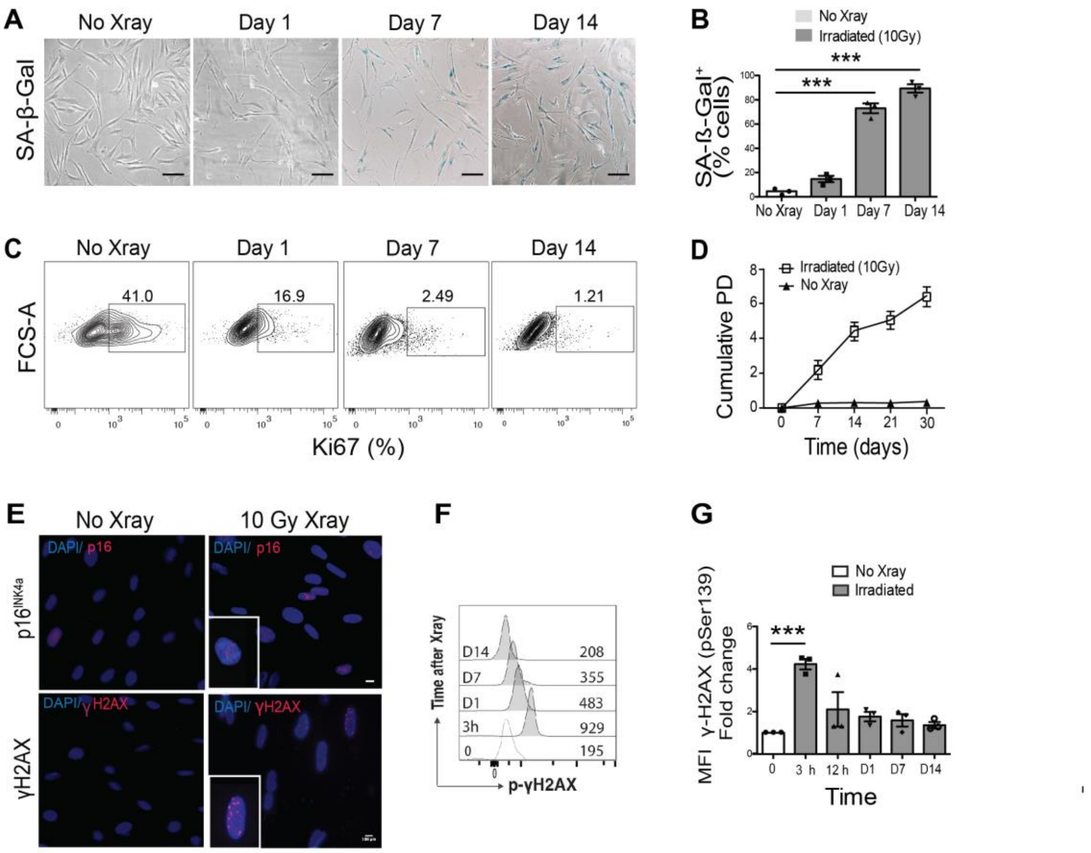

Supplementary Figure 1 - Ionizing radiation (IR) induces senescence in human fibroblasts. (**A**) Histochemical staining of irradiated fibroblasts for senescence-associated β-galactosidase (SA-β-Gal). Non-irradiated cells were used as controls (first panel). (**B**) Summary data obtained as in A) and presented as the percentage of SA-β-Gal^{+} cells (n=3). (**C**) FACS analysis of Ki67 expression at the indicated intervals after IR. Numbers indicate percentages of Ki67^{+} cells. (**D**) Growth curve of irradiated (☐) and non-irradiated fibroblasts (Δ). Cumulative population doublings (cPD) were calculated as described in Methods (n=5). **(E)** Immunofluorescence staining of γH2AX (Ser139) and p16^{INK4a} of irradiated fibroblasts (day 14 after IR) compared to controls. **(F)** Time-course of γH2AX phosphorylation (Ser139) after IR, assessed by phospho-flow cytometry. Numbers indicated mean fluorescence intensity (MFI). **(G)** Summary data presented as MFI fold change compared to non-irradiated controls (*n*=3). Statistical analysis performed with repeated measures ANOVA with correction for multiple comparisons. Error bars represent mean + SEM. Scale bar = 100µm. *$p<0.05$, **$p<0.01$, ***$p<0.001$, ****$p<0.0001$

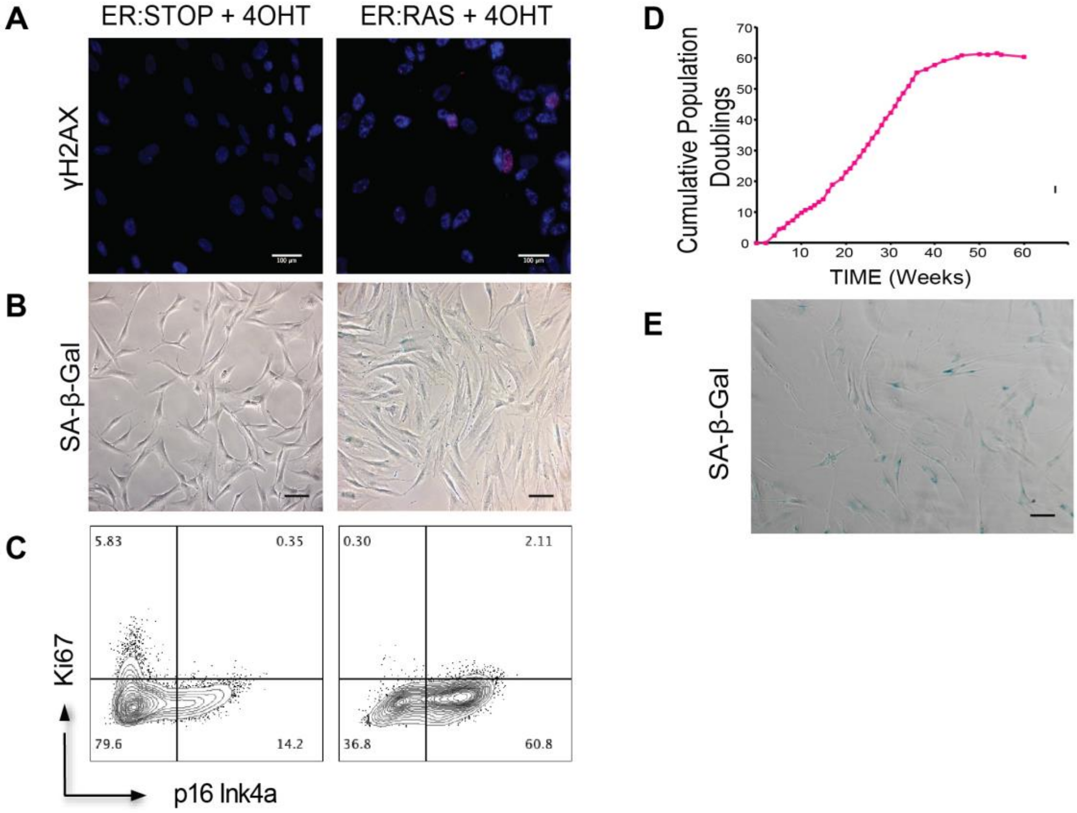

Supplementary Figure 2 – Oncogene-induced and replicative senescence models. IMR-90 cells infected with a lentivirus expressing *H-RAS*^{G12V} or a control vector (STOP) associated with tamoxifen-induced ER fusion protein were treated with 200 nM tamoxifen (4OHT) for 7 days and analysed for expression of senescence markers. (**A)** Immunofluorescence staining of γH2AX (Ser139); (**B)** Histochemical staining of SA-β-galactosidase activity; (**C)** Flow cytometry analysis of Ki67 and p16^{INK4a} expression, demonstrating increased expression of p16^{INK4a} and decreased Ki67 expression after activation of *H-RAS*^{G12V}. (**D**) Growth curve of primary human fibroblasts passaged until replicative senescence (plateau), confirmed by the presence of SA-β-galactosidase activity (**E**) in cells with cumulative population doublings (cPD) > 60. Scale bar = 100µm. Images are representative of 3 different experiments with similar findings.

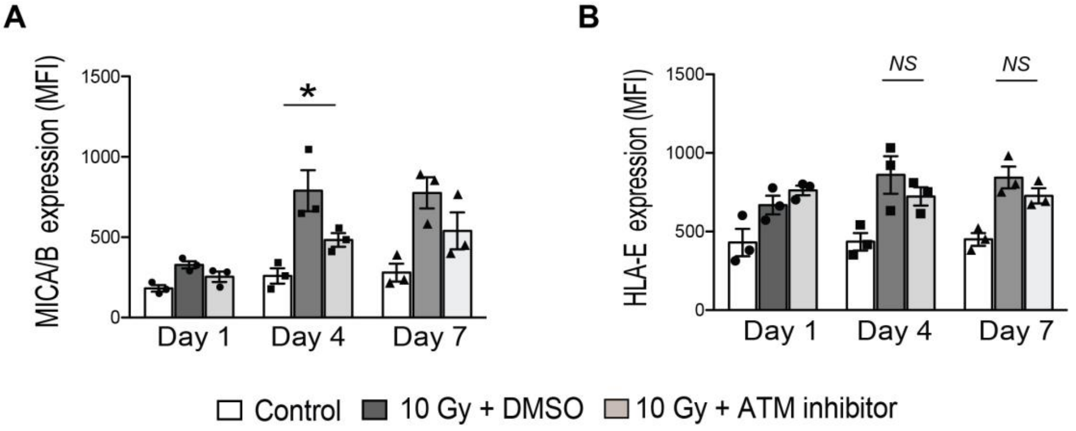

Supplementary Figure 3 – Effect of ATM inhibition on MICA/B and HLA-E expression. Primary human fibroblasts were treated with 10μM KU55933 12 hours before irradiation and continuously thereafter, over 7 days. Cells were harvested at the indicated times and compared to DMSO-treated and non-irradiated controls for cell surface expression of HLA-E and MICA/B by flow cytometry. Summary data (n=3) of the effect of ATM inhibition on MICA/B **(A)** and HLA-E expression **(B.**. Comparison between treated and control groups performed with the Mann-Whitney *U* test. Data presented as means ± SEM. *$p<0.05$, **$p<0.01$, ***$p<0.001$, ****$p<0.0001$

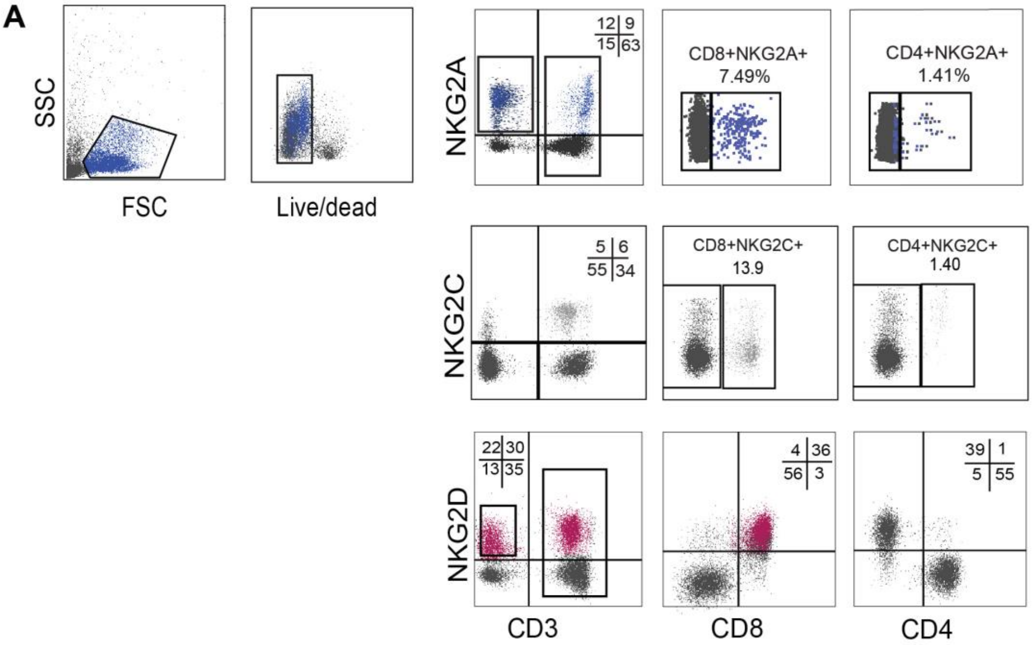

Supplementary Figure 4 – FACS sequential gating strategy used in Figure 3 A-C. Gating strategy used to analyse the distribution of NKG2A+ (top panel), NKG2C+ (middle panel) and NKG2D+ cells (bottom panel) in each compartment – CD3-negative, CD3-positive, CD4-positive and CD8-positive (n=27; mean age 49.9; range, 27-83). Numbers indicate percentages of gated cells in each compartment.

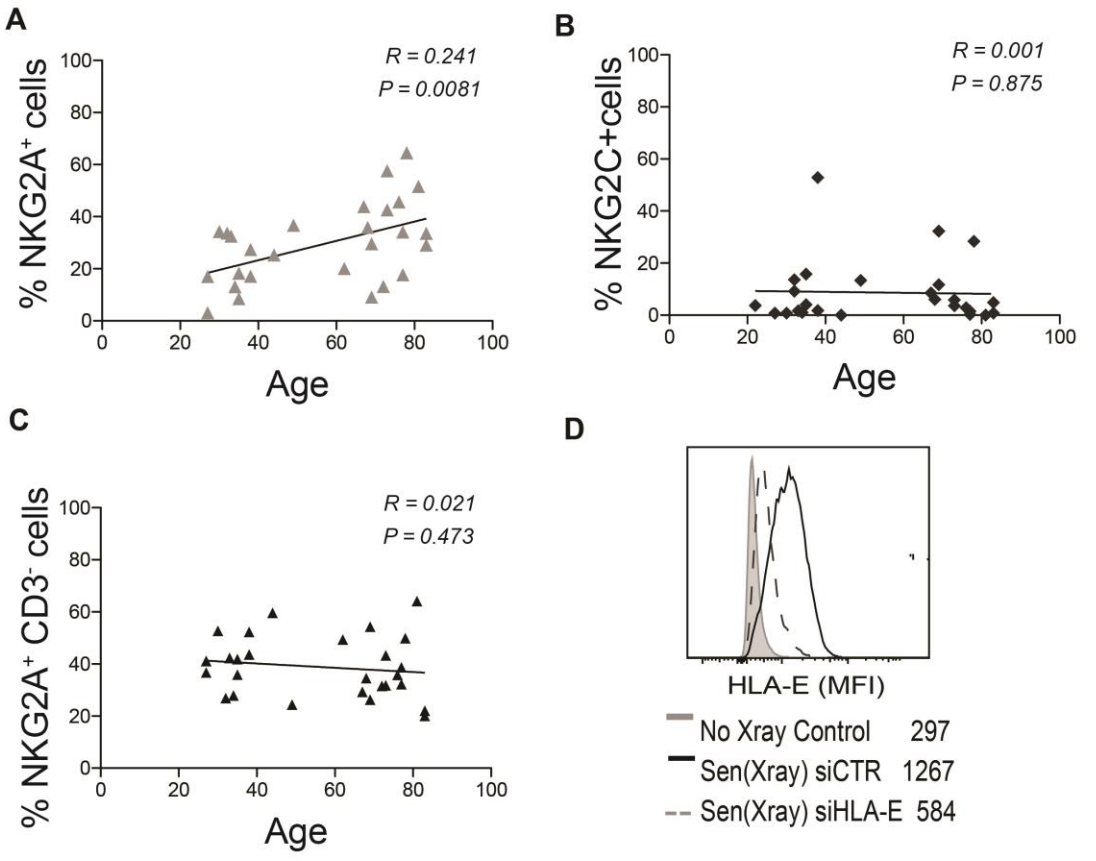

Supplementary Figure 5 – Correlation of NKG2A/C expression with age. Summary scatterplot of the correlation between age and the frequency of highly differentiated $CD8^+$ T cells expressing NKG2A **(A)** and **(B)** NKG2C, showing a significant increase of $CD8^+NKG2A^+$ cells with age, assessed by Spearman test (*n*= 27; mean age 49.9; range, 27-83). **(C)** Summary scatterplot of the correlation between age and percentage of $CD3^-$ cells expressing NKG2A, (*n*= 27; mean age 49.9; range, 27-83). **(D)** Normal and senescent fibroblasts were transfected with siRNA to HLA-E (sc-62470) or a control siRNA (sc-37007) and HLA-E expression was confirmed 36h after transfection using flow cytometry. Values indicate the Mean Fluorescence Intensity (MFI) of HLA-E (n=4).

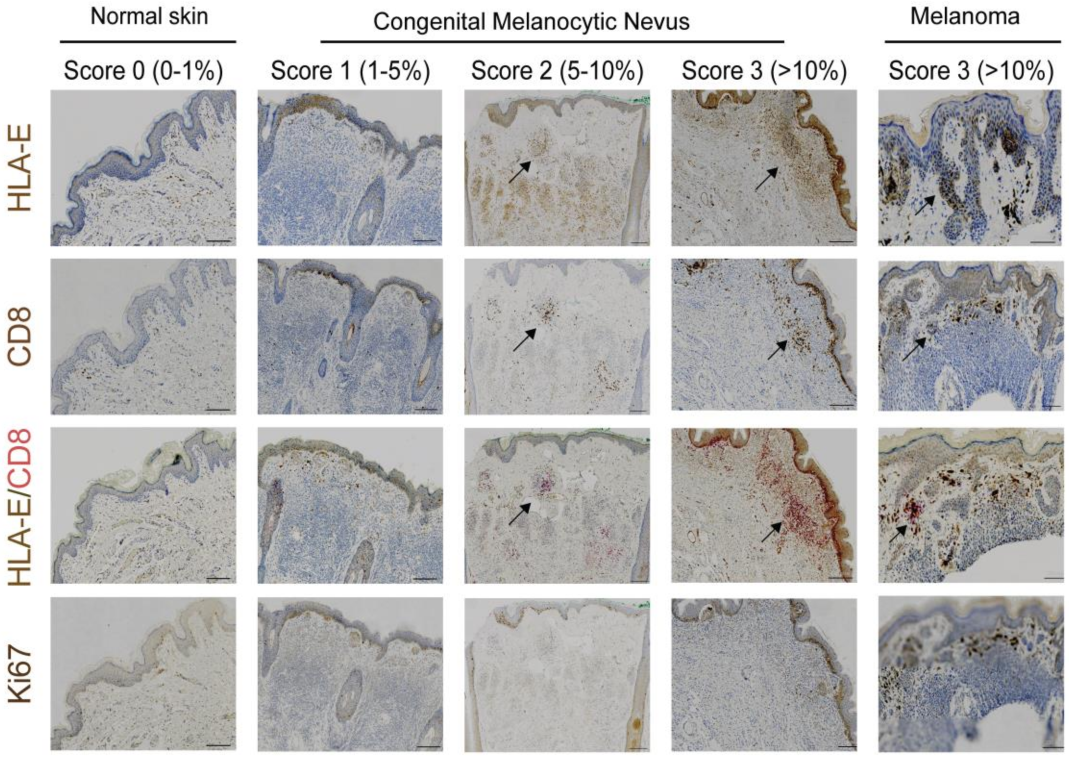

Supplementary Figure 6 - Immunohistochemical staining of human melanocytic nevi. Formalin-fixed paraffin-embedded tissue arrays of human melanocytic nevi were analysed by immunohistochemistry using HLA-E (MEM-E/02), CD8 and Ki67 specific antibodies. When double-staining for HLA-E and CD8, different chromogens reveal HLA-E (brown) and CD8 (red). Images are representative staining of normal skin, congenital melanocytic nevi and melanoma showing the different scores of intensity for HLA-E, based on the percentages of positive cells for HLA-E (indicated in brackets). Scale bar = 50 μm.

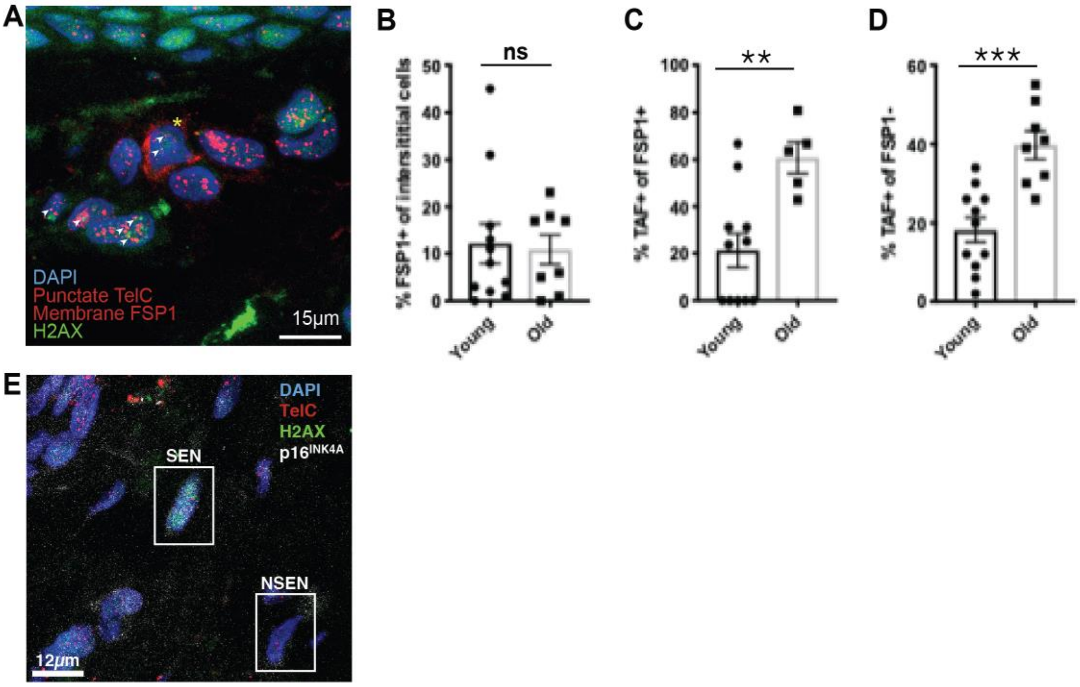

Supplementary Figure 7 - (A) Histological sections from skin punch-biopsies of young (<40 years) and old (>65 years) healthy donors were taken and stained for DAPI (blue), TelC (red punctate intranuclear), FSP1 (red membrane) and gammaH2AX (green). TAF are shown (white arrow heads) in $FSP1^+$ (yellow asterisk) and $FSP1^-$ interstitial cells. **(B)** Frequency of $FSP1^+$ interstitial cells in the dermis of young and old donors. **(C)** Frequency of TAF^+ cells amongst the $FSP1^+$ population of interstitial cells of the human dermis. **(D)** Frequency of TAF^+ cells amongst the $FSP1^-$ population of interstitial cells of the human dermis. **(E)** Histological sections from skin punch-biopsies were stained for DAPI (blue), TelC (red punctate intranuclear), γH2AX S139 (green) and $p16^{INK4A}$ (white). Telomere-associated γH2AX foci (TAF) are shown in non-senescent (NSEN) and senescent (SEN) cells in the dermis of human skin.

Supplementary Tables

	HLA-E Intensity Score	
	n	**%**
**Score 0 (0-1%)**	1	4.2
**Score 1 (1-5%)**	7	29.2
**Score 2 (5-10%)**	6	35.0
**Score 3 (>10%)**	10	41.7
Total	24	100

Supplementary Table 1 – HLA-E intensity score according to the percentage of HLA-E^{+} cells in human melanocytic nevi.

Antibody	Conjugate	Clone	Isotype	Manufacturer	Catalog #	Dilution
MHC-I	PE	W6/32	Mouse IgG2a	eBioscoence	12-9983	1:20
MHC-II	FITC	G46.6	Mouse IgG2a	BD Biosciences	556643	1:20
HLA-E	APC	3D12	Mouse IgG1	eBioscoence	17-99553	1:20
HLA-G	PE	87G	Mouse IgG2a	eBioscoence	17-9957	1:20
MICA/B	FITC	159207	Mouse IgG2a	R&D Systems	FAB13001	1:20
MICA/B	PE	6D4	Mouse IgG2b	BD Biosciences	558352	1:20
ULPB1	PE	170818	Mouse IgG2a	R&D Systems	FAB1380	1:20
ULBP 2,5,6	APC	165903	Mouse IgG2a	R&D Systems	FAB1298A	1:20
CD3	BUV 395	UCHT1	Mouse IgG1	BD	563546	1:100
CD4	PercP Cy 5.5	SK3	Mouse IgG1	Biolegend	344608	1:50
CD8	BV 421	RPA-T8	Mouse IgG1	Biolegend	301036	1:50
CD27	BV 786	L128	Mouse IgG1	BD	563327	1:50
CD28	BV 510	T44	Mouse IgG1	Biolegend	302936	1:50
CD45RA	BV 605	HI100	Mouse IgG2b	Biolegend	304134	1:50
NKG2A	AF 700	131411	Mouse IgG2a	R&D Systems	FAB 1059N	1:20
NKG2C	APC	134591	Mouse IgG1	R&D Systems	FAB138A	1:20
NKG2D	PE	149810	Mouse IgG1	R&D Systems	FAB139P	1:20

Supplementary Table 2 – Antibodies used in flow cytometry.

CMN label	Area (µm²)	# Nucleus(blue)	# Nucleus(Red)	Red bordering Brown (CD8 bordering HLA-E)	# Nucleus(Brown)	Area IHC_Brown(µm²)	#Red/#Blue*100 (CD8/total nucleous)	#Brown/#Blue*100 (HLAe/nt)	Red bordering Brown/total red
MDI	2409184	11107	507	405	658	61662	4.56	5.92	79.88
MDI	5294618	16327	1427	254	5155	495109.5	8.74	31.57	17.80
MDI	4884588	13731	879	724	8125	450164.5	6.40	59.17	82.37
MDI	2536287	9366	185	52	1111	159056.25	1.98	11.86	28.11
MDI	668465	644	82	35	249	59336	12.73	38.66	42.68
MDI	3309243	18117	167	107	1439	107703	0.92	7.94	64.07
MDI	2789191	10397	520	176	1211	86756.5	5.00	11.65	33.85
MDI	1865116	6399	43	42	435	25136.5	0.67	6.80	97.67
MDI	469211	1617	90	21	53	2265.75	5.57	3.28	23.33
MDI	2847470	7633	544	200	2444	152364	7.13	32.02	36.76
MDI	902587	2885	105	58	177	12452.25	3.64	6.14	55.24
MDI	3403055	14296	606	312	1641	148881.75	4.24	11.48	51.49
MDI	937157	3794	164	78	328	15894.75	4.32	8.65	47.56
MDI	2498848	9442	409	145	1989	113468.25	4.33	21.07	35.45
SNDI	1914257	9160	599	195	1026	91804.25	6.54	11.20	32.55
SNDI	2338713	13905	1024	467	556	141636.75	7.36	4.00	45.61
SNDI	1472323	3790	627	147	169	7365.5	16.54	4.46	23.44
SNDI	941658	7332	4	4	6	4922.25	0.05	0.08	100.00
SNDI	1417008	9293	541	377	524	57882.75	5.82	5.64	69.69
SNDI	1252299	9202	263	220	453	42254.75	2.86	4.92	83.65
SNDI	1190083	10202	101	67	427	45777.5	0.99	4.19	66.34
SNDI	1263369	7964	175	148	248	51623	2.20	3.11	84.57
SNDI	1575296	11112	301	249	407	80523.75	2.71	3.66	82.72
SNDI	1259527	5114	596	288	589	63354.5	11.65	11.52	48.32
**Mean**	**2059981.38**	**8867.88**	**414.96**	**198.79**	**1225.83**	**103224.83**	**5.29**	**12.87**	**55.55**
**STDEV**	**1244673.12**	**4507.07**	**348.91**	**169.43**	**1834.12**	**123721.85**	**4.02**	**14.07**	**24.87**

Supplementary Table 3 – Quantification of HLA-E^{+} and CD8^{+} cells by digital image analysis of human melanocytic nevi stained using immunohistochemistry.
