# Supplementary material for: Senescent cells evade immune clearance via HLA-E-mediated NK and CD8+ T cell inhibition
Source: Nat Commun. 2019 Jun 3;10:2387. doi: 10.1038/s41467-019-10335-5 (PMC6547655; doi:10.1038/s41467-019-10335-5)
Supplement: Supplementary file 2 — Reporting Summary [file 41467_2019_10335_MOESM2_ESM.pdf]

nature research

Corresponding author(s): Arne N Akbar

Last updated by author(s): Apr 15, 2019

Reporting Summary

Nature Research wishes to improve the reproducibility of the work that we publish. This form provides structure for consistency and transparency in reporting. For further information on Nature Research policies, see Authors & Referees and the Editorial Policy Checklist.

Statistics

For all statistical analyses, confirm that the following items are present in the figure legend, table legend, main text, or Methods section.

n/a	Confirmed	
☐	☒	The exact sample size (*n*) for each experimental group/condition, given as a discrete number and unit of measurement
☐	☒	A statement on whether measurements were taken from distinct samples or whether the same sample was measured repeatedly
☐	☒	The statistical test(s) used AND whether they are one- or two-sided<br>*Only common tests should be described solely by name; describe more complex techniques in the Methods section.*
☐	☒	A description of all covariates tested
☐	☒	A description of any assumptions or corrections, such as tests of normality and adjustment for multiple comparisons
☐	☒	A full description of the statistical parameters including central tendency (e.g. means) or other basic estimates (e.g. regression coefficient) AND variation (e.g. standard deviation) or associated estimates of uncertainty (e.g. confidence intervals)
☐	☒	For null hypothesis testing, the test statistic (e.g. *F*, *t*, *r*) with confidence intervals, effect sizes, degrees of freedom and *P* value noted<br>*Give P values as exact values whenever suitable.*
☒	☐	For Bayesian analysis, information on the choice of priors and Markov chain Monte Carlo settings
☒	☐	For hierarchical and complex designs, identification of the appropriate level for tests and full reporting of outcomes
☒	☐	Estimates of effect sizes (e.g. Cohen's *d*, Pearson's *r*), indicating how they were calculated

Our web collection on statistics for biologists contains articles on many of the points above.

Software and code

Policy information about availability of computer code

Data collection	Flow cytometry samples were acquired on a LSR II flow cytometer (BD Biosciences); Tissue array slides were scanned on a LEICA SCN400F digital slide scanner (Leica Microsystems). Imaging of TAF foci was performed using a Leica SPE2 confocal microscope (Leica Microsystem). Imaging consisted of obtaining Z-stacks with a step-size of 0.5µm.
Data analysis	Flow cytometry data analysed using FlowJo software (TreeStar). Tissue array images were analysed on the SlidePath Digital Image Hub (Leica) with Definiens Tissue Studio 3.6 (Definiens AG). Protein bands in western blots were quantified using ImageJ software. Analysis of TAF foci was performed using Fiji image analysis software (Fiji.sc). Statistical analysis was performed using Prism (GraphPad Software).

For manuscripts utilizing custom algorithms or software that are central to the research but not yet described in published literature, software must be made available to editors/reviewers. We strongly encourage code deposition in a community repository (e.g. GitHub). See the Nature Research guidelines for submitting code & software for further information.

Data

Policy information about availability of data

All manuscripts must include a data availability statement. This statement should provide the following information, where applicable:

- Accession codes, unique identifiers, or web links for publicly available datasets
- A list of figures that have associated raw data
- A description of any restrictions on data availability

Provide your data availability statement here.

Field-specific reporting

Please select the one below that is the best fit for your research. If you are not sure, read the appropriate sections before making your selection.

☒ Life sciences ☐ Behavioural & social sciences ☐ Ecological, evolutionary & environmental sciences

For a reference copy of the document with all sections, see nature.com/documents/nr-reporting-summary-flat.pdf

Life sciences study design

All studies must disclose on these points even when the disclosure is negative.

Sample size	Samples sizes were different for each experiment according to availability of samples. Sample size is stated for each experiment.
Data exclusions	There were no data exclusions.
Replication	Experiments were repeated at least 3 times for reproducibility of data.
Randomization	This study was not a randomized control study.
Blinding	Blinding was not relevant to this study.

Reporting for specific materials, systems and methods

We require information from authors about some types of materials, experimental systems and methods used in many studies. Here, indicate whether each material, system or method listed is relevant to your study. If you are not sure if a list item applies to your research, read the appropriate section before selecting a response.

Materials & experimental systems

n/a	Involved in the study	
☐	☒	Antibodies
☐	☒	Eukaryotic cell lines
☒	☐	Palaeontology
☐	☒	Animals and other organisms
☐	☒	Human research participants
☒	☐	Clinical data

Methods

n/a	Involved in the study	
☒	☐	ChIP-seq
☐	☒	Flow cytometry
☒	☐	MRI-based neuroimaging

Antibodies

Antibodies used	Staining for telomere-associated ɣH2AX foci (TAF) was done with ɣH2AX (Ser139, Cell Signaling #9718, 1:250), p16INK4a (Abcam, ab108349, 1:100 or Sigma SAB5300499, 1:100) and HLA-E (clone 3D12, eBioscience, 1:100). Primary antibodies for immunoblotting were rabbit polyclonal anti-histone γH2A.X (pS139), anti-Hsp27 (pS78), anti-p38 MAPK (pThr180/Tyr182), anti-p53 and anti–GAPDH (all from Cell Signalling). For HLA-E expression we used mouse monoclonal anti-HLA-E (MEM-E/02, Santa Cruz Biotechnology). All primary antibodies were used at a dilution of 1:1000. Flow cytometric analysis of surface expression of MHC molecules was performed after a 30-minute incubation at 4° C in the presence of saturating concentrations of antibodies (Supplementary Table 2) and a live/dead stain. Proliferation was assessed by staining for Ki67 (mouse Anti-Human Ki-67 set, BD Biosciences), p16Ink4a expression was performed using PE Mouse anti-human p16 set (BD Biosciences, 556561). For the detection of γH2AX (Ser139) we used phosphoflow cytometry after fixation (10 min at 37 °C with Cytofix Buffer), permeabilisation (30 minutes at 4 °C with ice-cold Perm Buffer III), washing (twice with Stain Buffer, all from BD Biosciences) and incubation for 30 minutes at room temperature with Alexa Fluor 488-conjugated antibody to γH2AX (Ser139) (clone 2F3; BioLegend).
Validation	All antibodies used were previously validated by the manufacturers. HLA-E (MEM-E/02, Santa Cruz Biotechnology) was previously validated for western blotting as described in "Menier, C. et al. Characterization of monoclonal antibodies recognizing HLA-G or HLA-E: new tools to analyze the expression of nonclassical HLA class I molecules. Human immunology 64, 315-326 (2003)". All antibodies were optimized and titrated with appropriate positive and negative controls (isotype controls).

Eukaryotic cell lines

Policy information about cell lines

Cell line source(s)	Primary human fibroblasts were derived from human healthy volunteers after written informed consent. IMR90 ER:RAS and IMR90 ER:STOP cell lines were a gift from Dr. Juan Carlos Acosta from the MRC Institute of Genetics & Molecular Medicine.

	(Edinburgh, UK).
Authentication	None of the cell lines were authenticated
Mycoplasma contamination	We regularly check our cell lines for Mycoplasma contamination and they were negative.
Commonly misidentified lines (See ICLAC register)	*Name any commonly misidentified cell lines used in the study and provide a rationale for their use.*

Animals and other organisms

Policy information about studies involving animals; ARRIVE guidelines recommended for reporting animal research

Laboratory animals	p16-3MR mice were generated and bred in house from C57BL/6J male mus musculus background in AALAC-accredited Buck Institute for Research on Aging animal facility.
Wild animals	The study did not involve wild animals.
Field-collected samples	The study did not involve field-collected samples.
Ethics oversight	Experiments with p16-3MR mice were performed under protocols approved by the Buck Institute´s Animal Care and Use Committee.

Note that full information on the approval of the study protocol must also be provided in the manuscript.

Human research participants

Policy information about studies involving human research participants

Population characteristics	Blood and skin samples were obtained from healthy research volunteers: (n=27; mean age 49.9; range, 27-83).
Recruitment	Healthy volunteers were recruited from recruitment drives of healthy volunteers (with no gender or age discrimination) wishing to participate in research studies.
Ethics oversight	The study was approved by the Research Ethics Committee of University College London (06/Q0502/92) and the National Health Service Research Ethics Service (11/LO/1846). All volunteers provided written, informed consent.

Note that full information on the approval of the study protocol must also be provided in the manuscript.

Flow Cytometry

Plots

Confirm that:

- [ ] The axis labels state the marker and fluorochrome used (e.g. CD4-FITC).
- [x] The axis scales are clearly visible. Include numbers along axes only for bottom left plot of group (a 'group' is an analysis of identical markers).
- [x] All plots are contour plots with outliers or pseudocolor plots.
- [x] A numerical value for number of cells or percentage (with statistics) is provided.

Methodology

Sample preparation	Peripheral blood mononuclear cells (PBMC) were isolated by density gradient centrifugation (Ficoll–Hypaque, Amersham) from blood of healthy donors. Untouched NK and CD8+ T cells were freshly isolated by magnetic activated cell sorting (MACS, Miltenyi Biotec) using a negative selection procedure (>95% purity). For fibroblast staining, cells were washed in PBS and harvested after trypsin treatment, centrifuged at 1200 rpm for 10 minutes and resuspended in ice-cold PBS. Flow cytometric analysis of surface expression of MHC molecules was performed after a 30-minute incubation at 4° C in the presence of saturating concentrations of antibodies and a live/dead stain
Instrument	Samples were acquired on a LSR II flow cytometer (BD Biosciences).
Software	Data was analysed using FlowJo software (TreeStar).
Cell population abundance	NK cells usually account for 1-5% of total PBMCs whereas CD8+ T cells represent 5-10% of total leukocytes. A purity check (>95% purity) was performed using flow cytometry after cell sorting.
Gating strategy	For fibroblast staining. FSC/SSc gates were set at FSC 110/SSC192. Dead cells awere gated out with a DAPI live-dead stain.<br>For FACS staining of PBMCs, the lymphocyte population was identified on SSC-A/FSC-A plot then gated on SSC-A/viability dye to exclude dead cells from live cells. Gate on the SSC-A/CD3 to identify the CD3+ T cells and CD3' cells (where NK cells are

inlcuded). From the CD3+ gate, CD4+ and CD8+ populations were identified.

☒ Tick this box to confirm that a figure exemplifying the gating strategy is provided in the Supplementary Information.
